# Differences in Trochlear Morphology of a New Femoral Component Designed for Kinematic Alignment from a Mechanical Alignment Design

**DOI:** 10.3390/bioengineering11010062

**Published:** 2024-01-08

**Authors:** Maury L. Hull, Alexander Simileysky, Stephen M. Howell

**Affiliations:** 1 Department of Biomedical Engineering, Department of Mechanical Engineering, Department of Orthopaedic Surgery, University of California Davis, Davis, CA 95616, USA; 2 Department of Biomedical Engineering, University of California Davis, Davis, CA 95616, USA

**Keywords:** total knee arthroplasty, total knee replacement, patellar tracking, patellofemoral joint, patellofemoral instability, patellofemoral complications

## Abstract

Because kinematic alignment (KA) aligns femoral components in greater valgus and with less external rotation than mechanical alignment (MA), the trochlear groove of an MA design used in KA is medialized, which can lead to complications. Hence, a KA design has emerged. In this study, our primary objective was to quantify differences in trochlear morphology between the KA design and the MA design from which the KA design evolved. The KA and MA designs were aligned in KA on ten 3D femur-cartilage models. Dependent variables describing the morphology of the trochlea along the anterior flange, which extends proximal to the native trochlea, and along the arc length of the native trochlea, were determined, as was flange coverage. Along the anterior flange, the KA groove was significantly lateral proximally by 10 mm and was significantly wider proximally by 5 mm compared to the MA design (*p* < 0.0001). Along the arc length of the native trochlea, the KA groove was significantly lateral to the MA design by 4.3 mm proximally (*p* ≤ 0.0001) and was significantly wider proximally by 19 mm than the MA design. The KA design reduced lateral under-coverage of the flange from 4 mm to 2 mm (*p* < 0.0001). The KA design potentially mitigates risk of patellofemoral complications by lateralizing and widening the groove to avoid medializing the patella for wide variations in the lateral distal femoral angle, and by widening the flange laterally to reduce under-coverage. This information enables clinicians to make informed decisions regarding use of the KA design.

## 1. Introduction

Possible patellofemoral complications after total knee arthroplasty (TKA) are many and can be categorized into bone-related and soft tissue-related [1]. Bone-related complications include aseptic loosening, periprosthetic fracture, and bone loss, whereas soft tissue-related complications include patellar instability, patellar clunk, and extensor mechanism failure. These complications represent one of the major causes for revision surgeries [2,3]. Two key factors in the development of patellofemoral complications are alignment of the femoral component [2,4,5,6] and prosthetic trochlear morphology [7,8], both of which affect the position and orientation of the trochlear groove which guides patellar tracking during knee flexion.

**Figure 1:**
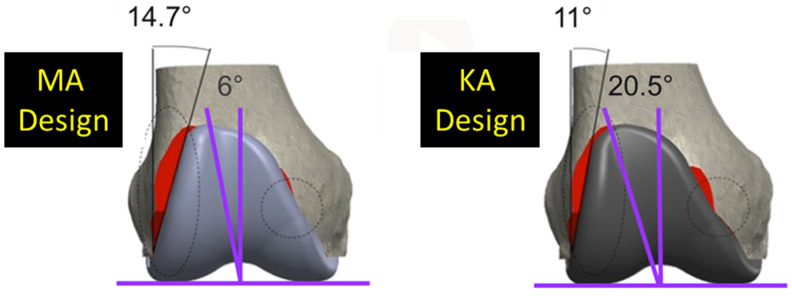
Coronal views of the of the MA design and the KA design. Two major design modifications were widening the trochlear groove from 6° to 20.5° and decreasing the angle of the lateral border from 14.7° to 11.0°. Red shows areas of resection uncovered.

Since two alignment philosophies for TKA are used and differ in their approach for aligning the femoral component, it is important to appreciate how the difference affects the trochlear morphology and, in turn, patellofemoral joint function. Kinematic alignment (KA) is an alignment philosophy which has emerged as an alternative to mechanical alignment (MA) [9]. MA aligns the femoral component so that the distal femoral joint line is perpendicular to the mechanical axis of the femur, and the internal-external (I-E) rotation is set according to one of several methods [10,11]. In contrast, KA aligns the femoral component to restore the native (i.e., pre-arthritic) distal and posterior femoral joint lines. KA sets the femoral component on average 2.2° [12] to 4.6° more valgus than MA [13]. And if I-E rotation in MA is set 3° external, which is common [10,11], then rotation in KA is 2.8° more internal than in MA [13]. As a result, for femoral components designed for MA but used in KA, the lateral reach of the flange might be reduced [14] and the trochlear groove medialized particularly proximally [13,15]. Medializing the groove can lead to patellar maltracking, and reducing the lateral reach can lead to under-coverage. Accordingly, at least one manufacturer to date has modified its MA femoral component design to generate a KA design with a redesigned flange, featuring a reduced angle of the lateral border and widened trochlea to prevent these undesirable effects (Figure 1).

The objectives were twofold. By aligning computer-aided design (CAD) models of femoral components in KA on highly accurate 3D femur-cartilage models of native limbs, the primary objective was to quantify differences in morphology between the new KA design and the original MA design. A secondary objective was to quantify differences in the morphology of the KA design from native. Collectively, these results would inform clinicians of the potential benefits of the KA design in mitigating undesirable effects of the MA design.

## 2. Materials and Methods

Healthy femurs from ten unpaired fresh-frozen human cadaveric lower limbs without evidence of prior fracture after review of a computer tomographic (CT) scan and without femoral articular wear at inspection during dissection were studied (median age: 75.5 years ranging from 51–94 years, 7 females and 3 males). High-accuracy 3D femur-articular cartilage models were created using a combination of CT scans performed with 0.625 mm slice thickness with the knee joint intact in conjunction with a laser scan for the articular surfaces with the knee joint disarticulated. Fiducial markers attached to the shaft were used to register the 3D bone models created from the CT scans with the 3D distal femur-articular cartilage models created from the laser scanner.

To align the femoral components in KA, 3D femur-cartilage models were projected in standard sagittal, coronal, and axial planes [13]. The same size femoral components were used for each 3D femur-cartilage model. The varus-valgus (V-V) rotation and proximal-distal (P-D) position were set coincident to the distal cartilage surfaces at 0° flexion, whereas the internal-external (I-E) rotation and anterior-posterior (A-P) position were set coincident to the posterior cartilage surfaces of the femur at 90° flexion. The flexion-extension (F-E) rotation of the femoral component was set parallel to the sagittal projection of the mechanical axis of the femur, and the medial-lateral (M-L) location was set by centering the femoral component. Femoral components were correctly sized when M-L overhang was 1 mm or less and the gap between the anterior flange and anterior resection was 1 mm or less.

**Figure 2:**
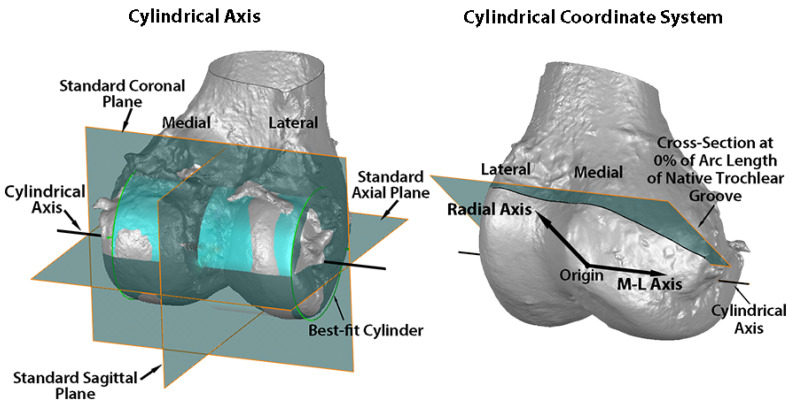
Images showing the standard planes and the relationship of the cylindrical axis with respect to the standard planes on a posterior oblique view of the 3D femur-cartilage model (**left**), and the origin, medial-lateral (M-L) axis, radial axis, and reference plane of the cylindrical coordinate system on an anterior oblique view of the 3D femur-cartilage model (**right**). The cylindrical axis (black line) passes through the center of a cylinder (green) best-fit to the central third of the cartilage on each femoral condyle (**left**). The origin of the cylindrical coordinate system was on the M-L axis (i.e., cylindrical axis) midway between the most medial and lateral points on the femoral condyles (**right**). A radial axis set at the proximal edge of the groove of the native trochlea defined the plane of the 0% cross-section along the arc length of the native trochlear groove. The relationship of the cylindrical axis and coordinate system to the prosthetic trochlea is not shown.

**Figure 3:**
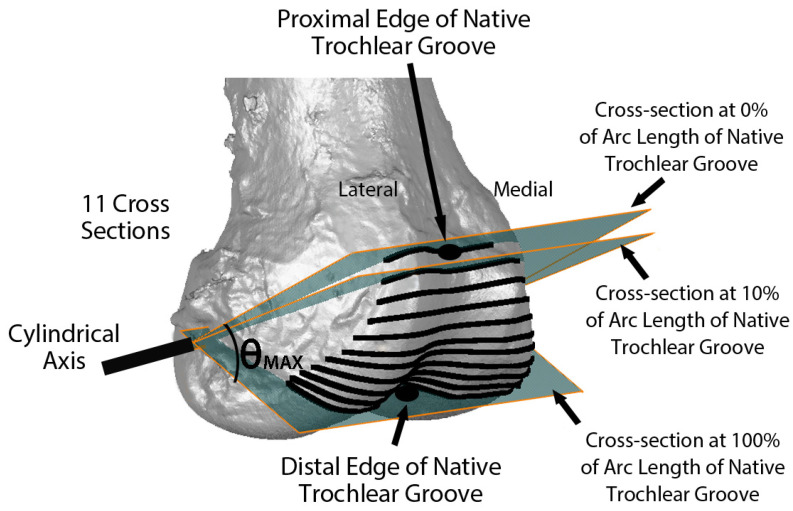
Image showing the relationship of eleven cross-sections along the arc length of the native trochlear groove with respect to the cylindrical axis on an oblique view of the 3D femur-cartilage model. The 0% cross-section was set coincident to the proximal edge of the trochlear groove, and the 100% cross-section was set at the most distal edge. Not shown are the projections of the cross-sections on the KA and MA prosthetic trochleas.

Variables describing the trochlear geometry were determined at specified cross-sections for each femoral component design when positioned on the 3D femur-cartilage models. The designs included the original MA design and the new KA design (GMK Sphere and SpheriKA, respectively, Medacta, Castel San Pietro, Switzerland). A cylindrical coordinate system was created by best-fitting a cylinder to the cartilage surfaces of femoral condyles of the 3D femur-cartilage model (Figure 2). Eleven cross-sections of the 3D femur-cartilage model were constructed at 10% increments along the arc length of the native trochlear groove by rotating about the cylindrical axis (Figure 3). These cross-sections were propagated onto the prosthetic trochlea.

**Figure 4:**
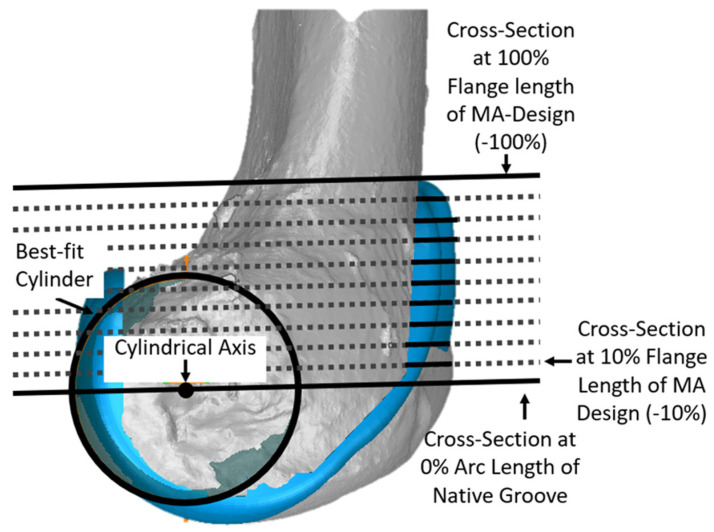
Additional cross-sections in planes parallel to the plane of the cross-section at 0% of arc length were used to describe the geometry of the anterior flange, which extends above the proximal edge of the groove. The −100% cross-section of the anterior flange corresponded to the most proximal point of the MA design. Although cross-sections in −10% increments are shown, analysis was performed only at cross-sections in −20% increments.

Since the anterior flange (defined as that portion of the femoral component covering the anterior resection) extends well above the most proximal cross-section of the native groove, additional cross-sections were added to describe the morphology of the anterior flange per se. For each size femoral component, cross-sections were parallel to the cross-section at 0% and normalized to the length of the original MA design. This added five additional cross-sections in −20% increments (Figure 4).

**Figure 5:**
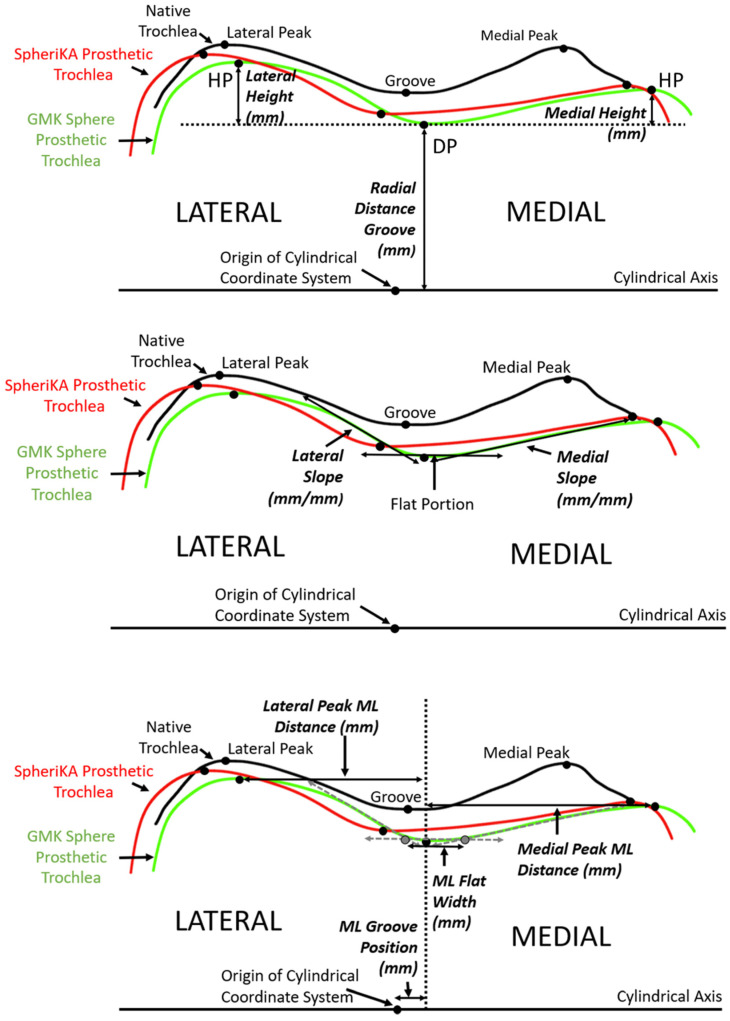
Diagrams of a representative cross-section of the distal femur showing the relationship between tracings of the articular surface of the native trochlea (black), the prosthetic trochlea for the MA design (green), and the prosthetic trochlea for the KA design (red). (**Top**) The landmarks of the deepest point (DP) of the groove and the highest point (HP) of the medial and lateral facets (only shown on the prosthetic trochlea for the MA design) were used to determine height variables, which included the radial distance of the groove and the heights of the medial and lateral peaks. (**Middle**) Medial and lateral slopes were tangents to the cross-section profile in the regions nearest the DP. (**Bottom**) Medial-lateral (ML) variables included the ML position of the groove, the ML flat width, and the medial and lateral peak ML distances from the DP.

A number of dependent variables quantified the geometry of each cross-section. The variables were grouped into three categories: (1) heights, (2) slopes, and (3) medial-lateral distances. To determine the height variables along the arc length, three points were identified at each cross-section (Figure 5 top). The three points were the point with the closest radial distance to the cylindrical axis and the points medial and lateral that were the farthest radial distance from the cylindrical axis. Using these three points, the three dependent variables were the radial distance of the groove and the medial and lateral heights. To determine the height variables along the anterior flange, the distances of the points medial and lateral that were farthest from the plane of the flange indicated the medial and lateral heights, respectively. To determine slope variables, lines were drawn tangent to the cross-section profiles in the medial and lateral regions nearest to the deepest point (Figure 5 middle). To determine medial-lateral (ML) distance variables, the ML distance of the DP (i.e., groove) from the origin of the cylindrical coordinate system was determined (Figure 5 bottom). From the DP, the ML distances to the medial and lateral peaks were measured. The final ML variable was the ML flat width; this width was the ML distance between points defined by the intersection of a line parallel to the cylindrical axis and tangent to the profile at the DP with the medial and lateral slopes.

Additional dependent variables for the anterior flange indicated coverage of the anterior resection. These included under-coverage and overhang of the femoral component both medially and laterally.

### Statistical Analysis

Descriptive statistics of the dependent variables were the mean ± standard deviation. Because significant and important interactions between the factors of knee condition (i.e., femoral component designs and native knee) and percent of arc length were evident, paired Student’s *t*-tests were performed at each percent of arc length along the native groove. The pairs were the KA and MA designs and the KA design and native. With a Bonferroni adjustment based on two paired comparisons at each cross-section along the arc length of the groove, a *p*-value of <0.025 indicated significance. For the anterior flange, paired *t*-tests also were performed at each percent of flange height with the pairs being the KA and MA designs. A *p*-value of <0.05 indicated significance for the latter paired *t*-test.

A power analysis confirmed that with ten femurs, differences in groove locations between designs of 2 mm, which do not cause adverse mechanical effects [16,17], could be detected with *α* = 0.05 and (1 − *β*) ≥ 0.80 using standard deviations of the differences in groove locations between alignment methods of 1.9 mm [18].

The repeatability (i.e., precision) of each dependent variable was quantified, and the repeatability and reproducibility were evaluated using an ICC analysis. Based on making five measurements of each of the dependent variables on five different specimens for each of three observers, the precisions were quantified by computing the pooled standard deviations (Table 1). Using a two-factor repeated measures ANOVA with mixed effects, the variance components for observer, 3D femur-cartilage model, and error were determined. These variance components were used to compute the intraobserver and interobserver ICC values (Table 1) [19]. An ICC value of >0.9 indicates excellent agreement, 0.75–0.90 indicates good agreement, 0.5–0.75 indicates moderate agreement, and 0.25–0.5 indicates fair agreement [20].

Following University of California policies, this study did not require institutional review board (IRB) approval because de-identified cadaveric specimens were used.

## 3. Results

### 3.1. Anterior Flange

**Figure 6:**
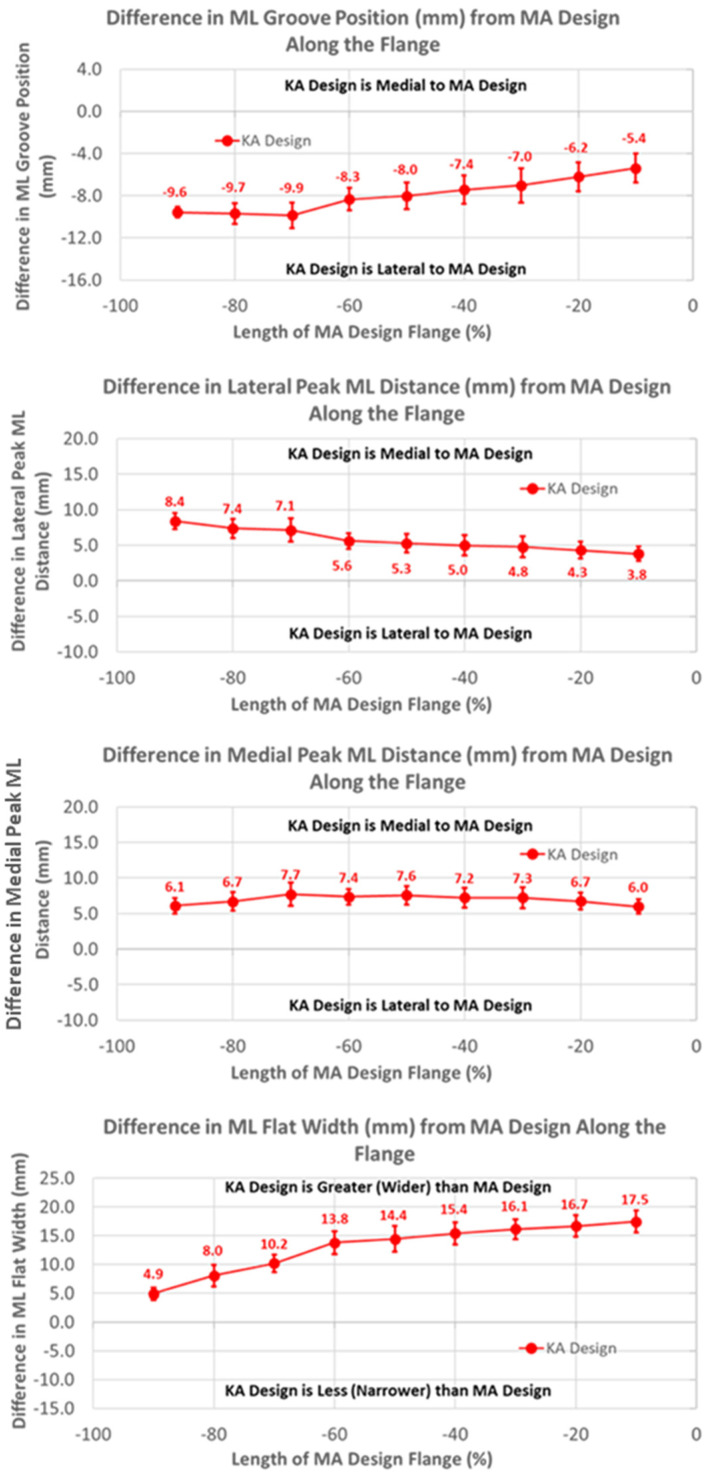
Graphs showing the mean ± one standard deviation for differences in medial-lateral variables between the KA and MA designs along the anterior flange at intervals from −100% (proximal) to 0% (distal) of the normalized length of the flange of the MA design.

The groove in the KA design was considerably more lateral than the MA design by 10 mm, proximally decreasing to about 5 mm distally over the length of the flange (Figure 6) (*p* < 0.0001). Differences in the ML distance of the lateral peaks from the MA design started at about 8 mm medial, proximally decreasing to about 4 mm distally (*p* < 0.0001). Considering that the magnitudes of the lateral differences of the groove were greater than the magnitudes of the medial differences in the lateral peaks, the absolute positions of the lateral peaks were shifted laterally about 2 mm relative to the lateral peaks of the MA design. Likewise, since the groove position of the KA design was shifted laterally and the medial peak was shifted medially, the net shift was about 4 mm lateral in the absolute position of the medial peak, proximally decreasing to 0 mm distally. Finally, the flat width of the KA design was considerably wider than the MA design, ranging from 5 mm proximally to 17 mm distally (*p* < 0.0001).

**Figure 7:**
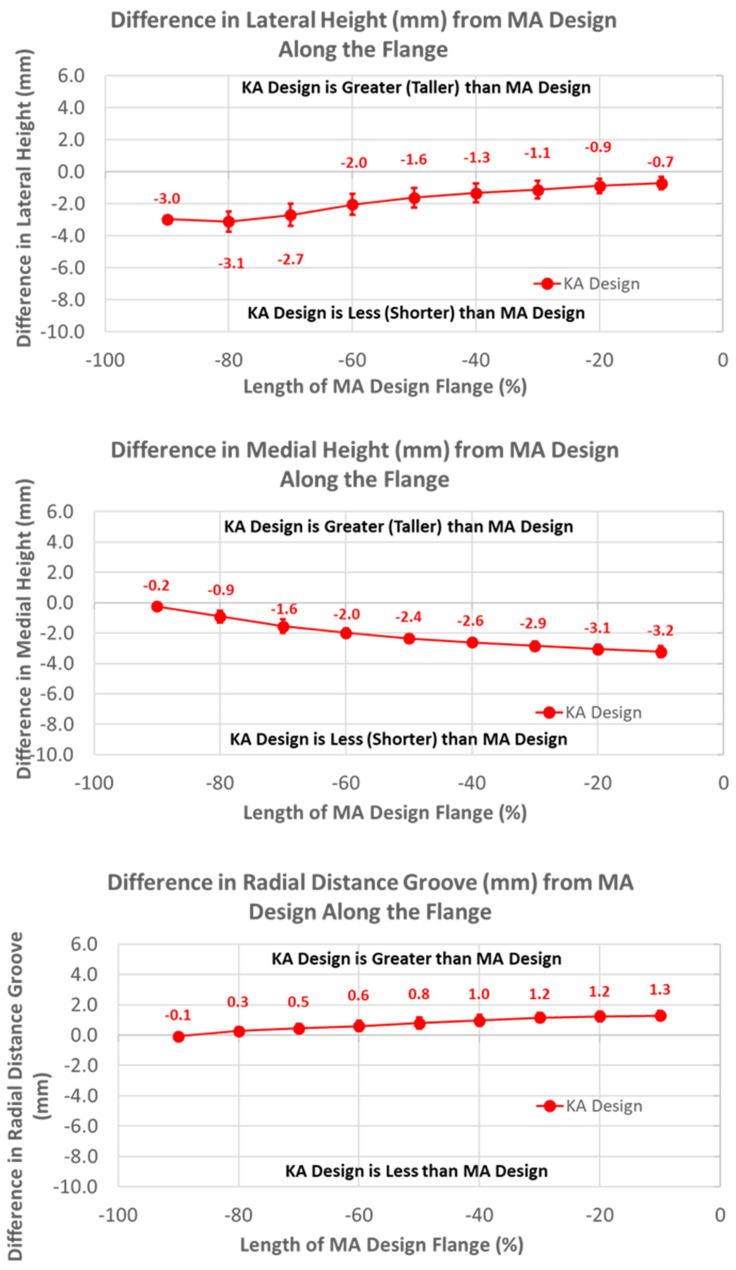
Graphs showing the mean ± one standard deviation for differences in lateral and medial heights and the radial distance between the KA and MA designs along the anterior flange at intervals from −100% (proximal) to 0% (distal) of the normalized length of the flange of the MA design.

The heights of the lateral and medial peaks with the KA design were consistently lower than the MA design (Figure 7) (*p* ≤ 0.0002). In addition, trends from the MA design differed, with the heights of the lateral and medial peaks steadily increasing and decreasing in magnitude, respectively, moving from proximal (i.e., −100%) to distal (i.e., 0%).

**Figure 8:**
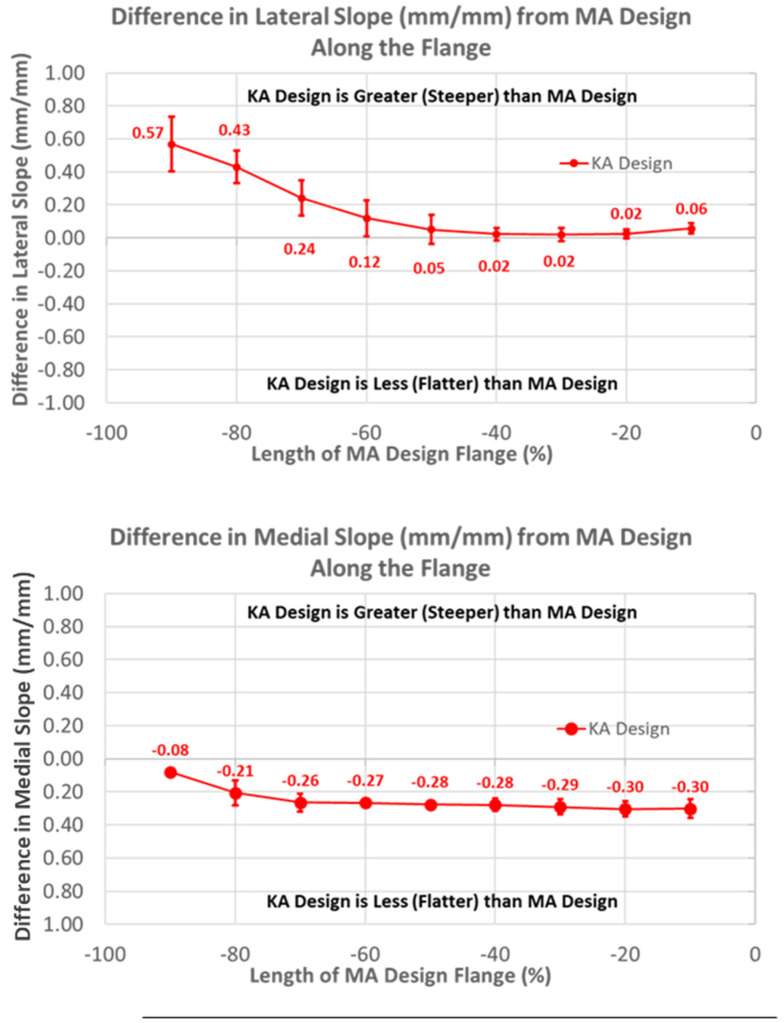
Graphs showing the mean ± one standard deviation for differences in lateral and medial slopes between the KA and MA designs along the anterior flange at intervals −100% (proximal) to 0% (distal) of the normalized length of the flange of the MA design.

Likewise, patterns of slope variables for the KA design patterns differed from the MA design for lateral versus medial slopes (Figure 8). The lateral slope was steeper proximally by 0.57 mm/mm (*p* < 0.0001) and steadily decreased, becoming equal to the MA design at −40% flange height (*p* = 1.0000), whereas the medial slope was less than the MA design over the full flange height (*p* < 0.0001).

**Figure 9:**
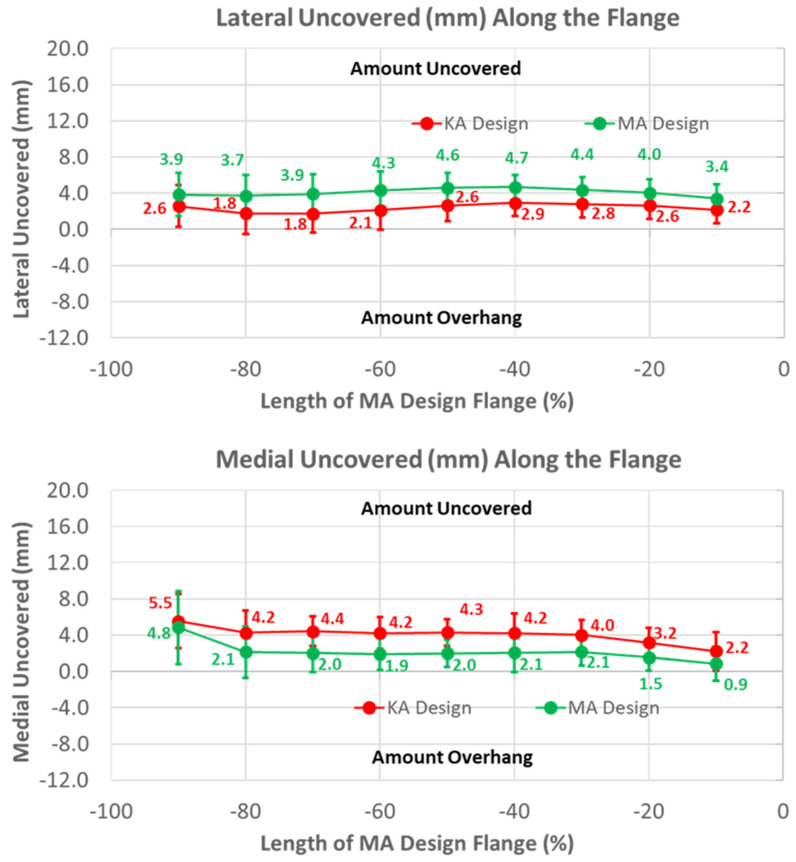
Graphs showing the mean ± one standard deviation of the coverage of the lateral and medial regions of the anterior resection for the KA and MA designs along the anterior flange at intervals from −100% (proximal) to 0% (distal) of the normalized length of the flange of the MA design.

Under-coverage laterally was lower for the KA than the MA design (Figure 9) (*p* < 0.0001). Under-coverage for the MA design averaged about 4 mm, whereas this was reduced to about 2.5 mm for the KA design. In contrast, the KA design had greater under-coverage medially than the MA design. Under-coverage for the MA design averaged about 2 mm, whereas under-coverage for the KA design averaged about 2 mm greater (*p* < 0.0001).

### 3.2. Arc Length of Native Groove: KA versus MA

**Figure 10:**
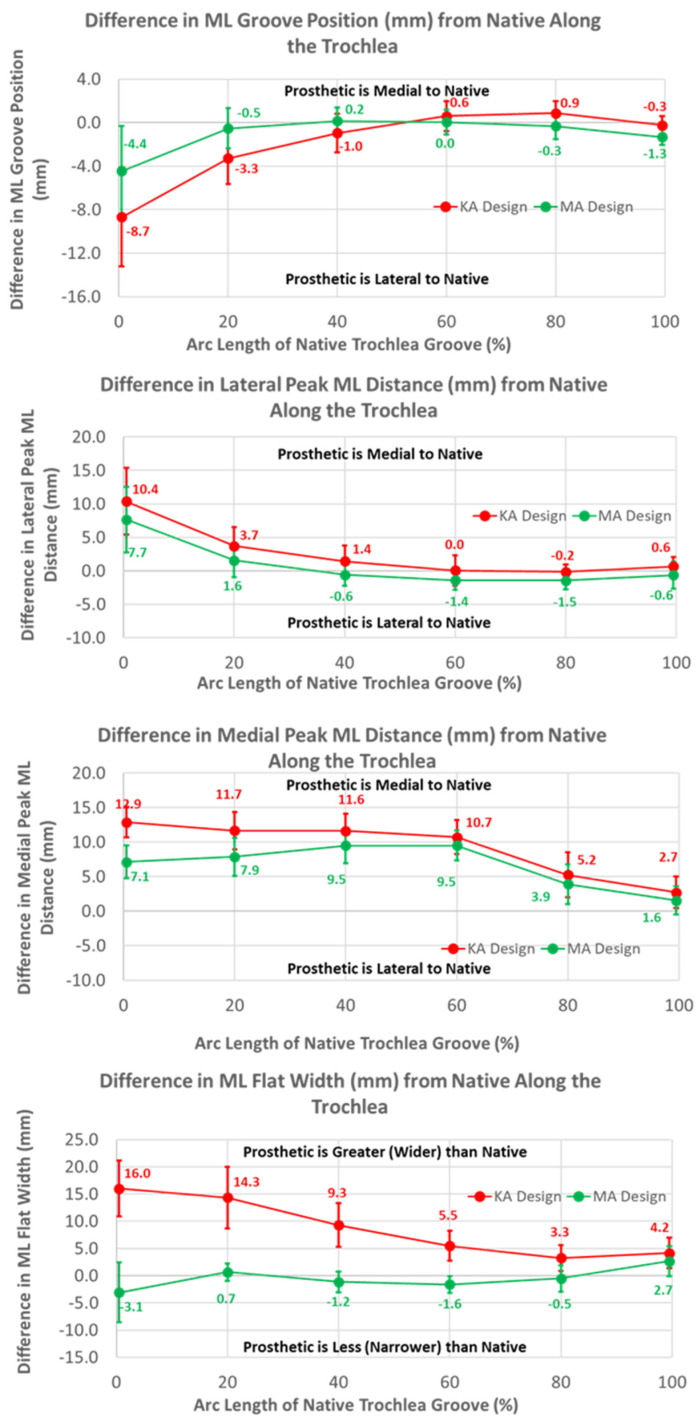
Graphs showing the mean ± one standard deviation for differences in medial-lateral variables between the native knee and the KA and MA designs along the native trochlear groove at intervals from 0% to 100% of normalized length of the native groove.

The ML variables differed between the KA and MA designs (Figure 10). Over the arc length range from 0% to 40%, the ML position of the KA groove was significantly lateral to that of the MA groove by 4.3 mm at 0%, gradually decreasing to 1.2 mm at 40% (*p* ≤ 0.0054). Differences in the ML distances to the peaks referenced to the DPs were greatest at 0% arc length, with the KA design being more medial by 2.7 mm (*p* < 0.0001) and 5.8 mm (*p* = 0.0201) for the lateral and medial peaks, respectively, than the MA design (Figure 10). The ML flat width was significantly wider for the KA than the MA design by about 19 mm proximally, and this difference progressively decreased distally but remained statistically significant (*p* ≤ 0.0176).

**Figure 11:**
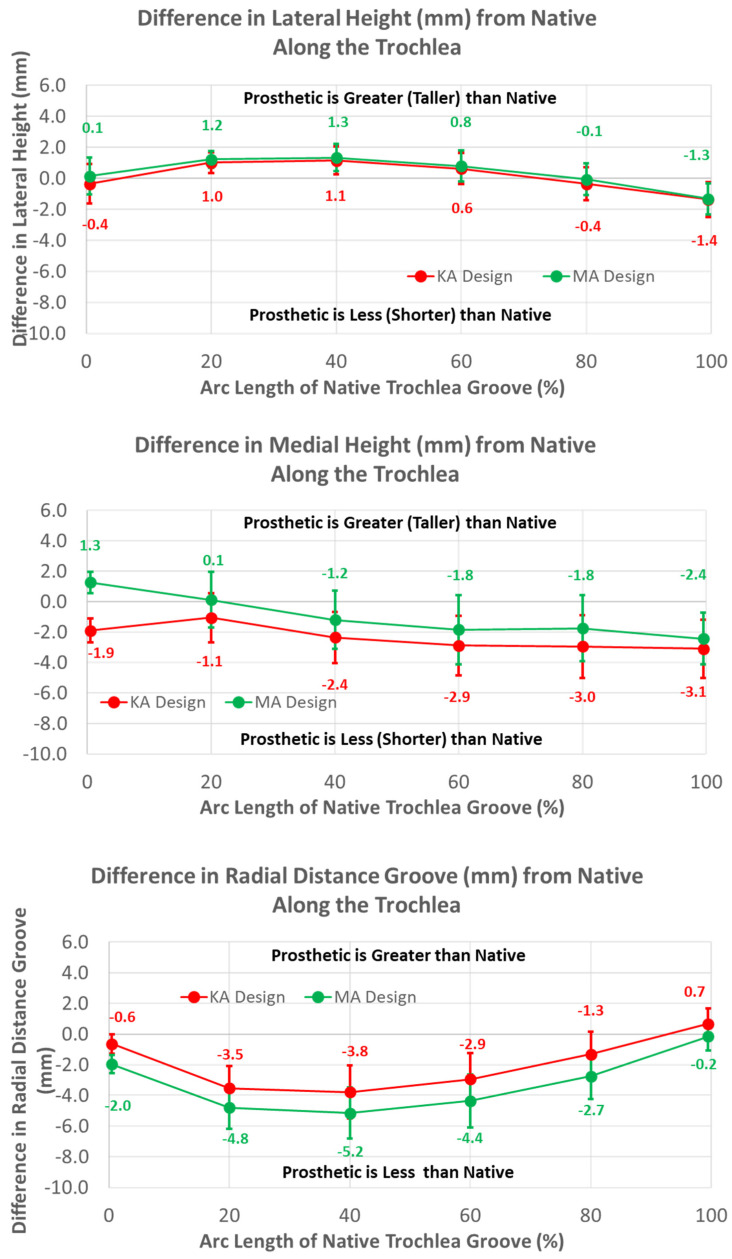
Graphs showing the mean ± one standard deviation for differences in medial and lateral heights and the radial distance between the native knee and the KA and MA designs along the native trochlear groove at intervals from 0% to 100% of normalized length of the native groove.

Regarding height variables, the groove of the KA design was closer to the cylindrical axis than the MA design by about 1.5 mm at most arc length percentages (*p* < 0.0001) (Figure 11). Differences in heights referenced to the deepest points between the MA and KA designs were apparent for medial but not lateral peaks; the height of the medial peak in the KA design was lower than the MA design over most of the arc length (*p* ≤ 0.0092).

**Figure 12:**
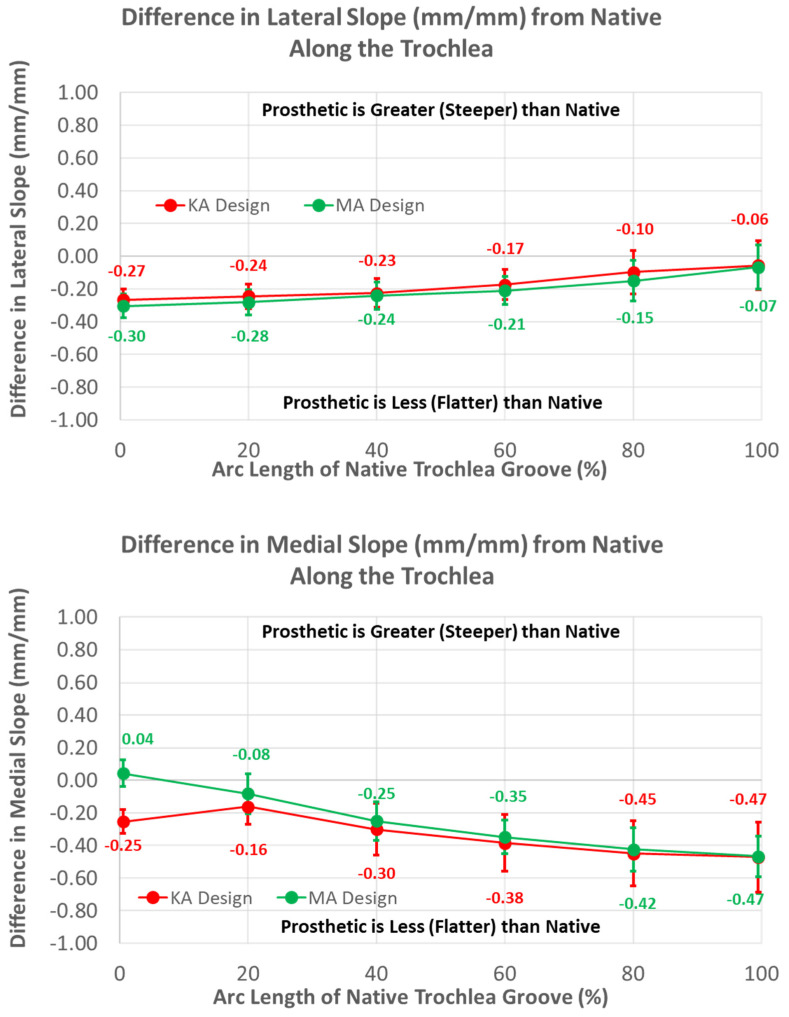
Graphs showing the mean ± one standard deviation for differences in lateral and medial slopes between the native knee and the KA and MA designs along the native trochlear groove at intervals from 0% to 100% of normalized length of the native groove.

Medial and lateral slopes compared closely between the two femoral component designs (Figure 12).

### 3.3. Arc Length of Native Groove: KA versus Native

The mean medial-lateral locations of the KA groove were significantly more lateral than the native knee at 0% (8.7 mm, *p* = 0.0002) and 20% (3.3 mm, *p* = 0.0018) of the arc length, and converged closely to native at 60% arc length (*p* = 0.1948). Noting that a lateral shift of the groove is offset by a medial shift of the lateral peak relative to the groove, the absolute ML position of the lateral peak for the KA design was 4 mm medial to the native peak. In contrast, a lateral shift of the groove in conjunction with a medial shift of the medial peak is additive, in which case the absolute ML position of the medial peak was about 19 mm medial to the native peak at 0% arc length. The ML flat width was significantly wider for the KA than native by about 16 mm proximally, and this difference progressively decreased distally but remained statistically significant (*p* ≤ 0.0011).

The mean radial locations of the KA groove were fewer than native over the majority of the arc length (*p* ≤ 0.0200). The height of the medial peak in the KA design deviated from native by about 3 mm at 40% arc length and beyond (*p* ≤ 0.0017). The height of the lateral peak also deviated from native particularly at 20% and 40% of the arc length (*p* ≤ 0.0031).

The greatest magnitude difference in lateral slope from native occurred at 0% arc length (*p* < 0.0001), and this difference gradually decreased, with increasing arc length becoming non-significant at 100% (*p* = 0.2669). In contrast, the smallest magnitude difference in the medial slope occurred at 0% arc length (*p* = 0.0290), and the magnitude progressively increased with increasing arc length, reaching a maximum at 100% (*p* < 0.0001).

## 4. Discussion

Because femoral components designed for MA but aligned in KA medialize the trochlear groove particularly proximally, a KA femoral component design has emerged to avoid patellar maltracking and increase coverage laterally of the anterior resection. In assessing differences in morphology between the KA design, the MA design, and the native knee, the key findings were as follows: (1) the trochlear groove of the KA design was shifted significantly laterally relative to the MA design and the native knee, (2) the trochlea of the KA design was significantly widened relative to the MA design and the native knee, and (3) lateral under-coverage of the flange with the KA design was significantly less than the MA design.

The finding that the trochlear groove of the KA design along the anterior flange and the native arc length was significantly more lateral proximally than the MA design is indicative of the design strategy. When the knee is straight, the distance from the distal femoral articular surface to the most proximal cross-section of the flange (i.e., −100%) is about 60 mm. The lateral distal femoral angle varies from 79.5° to 94.5° with a mean of 87° [21]. Angles less than 90° would medialize the trochlear groove relative to MA. A mean 3° valgus rotation of the femoral component in KA relative to MA would shift the proximal groove medially about 3 mm. However, the groove of the KA design was shifted laterally about 10 mm at −100% (Figure 6). This shift accounts for the variability in the lateral distal femoral angle where a 79.5° angle would require a lateral shift of 10 mm. Considering that the more lateral location of the KA groove was greatest at −100% and gradually aligned with the medial-lateral location of the MA design groove at 60% of the native arc length (Figure 10), the KA design potentially reduces the risk of patellofemoral instability over the full range of lateral distal femoral angles. To determine whether this potential is realized in practice, clinical studies with patient follow-up and/or biomechanical studies of patellofemoral joint function are warranted.

To shift the patellar groove laterally in the anterior flange by 10 mm proximally, the flange was necessarily widened laterally. A benefit of the widening was that under-coverage laterally was significantly reduced (Figure 9). A previous study used a different femoral component design with the same trochlear groove angle relative to vertical aligned in KA and MA, and found that the lateral under-coverage of the KA-aligned component was 3 mm on average [14]. This is consistent with our results and those of another study [22], both of which showed that under-coverage with the KA design aligned in MA was 4 mm on average (Figure 9), and emphasizes the need to widen the flange for KA femoral components to reduce under-coverage.

The mean radial location of the groove in the KA design was less recessed with respect to the native groove than the MA design but only by about 1.5 mm (Figure 11). This is interesting because the radial location of the KA-prosthetic groove remained less recessed than native over most of the native arc length and more than 3 mm less recessed in the 20% to 50% range of arc length (Figure 11). It is curious as to why the groove was recessed relative to native in the KA design. Assuming that the patella is resurfaced, the complications associated with under-stuffing/overstuffing the patellofemoral joint must be considered. Under-stuffing is undesirable because this can compromise the extensor mechanism. Under-stuffing decreases the moment arm of the quadriceps muscle force, thus increasing the quadriceps force needed to develop an extension moment and increasing the patellofemoral joint compression force. This increase in the joint compression force could cause anterior knee pain, a common complication with patellar resurfacing [1]. However, overstuffing the prosthetic patellofemoral joint should be avoided, since this can lead to other complications such as decreased flexion [17] and lateral patellar maltracking due to tightening of the lateral retinaculum with a concomitant increased risk of lateral patellar subluxation [23,24]. By maintaining a groove which is recessed more than native, the risk of complications due to overstuffing is mitigated rather than under-stuffing. Because one of these complications is lateral patellar subluxation, the decreasing risk of this complication parallels the redesigned medial-lateral location of the groove, which mitigates the risk of this complication.

A final design feature which merits discussion concerns the height of the anterior flange. The anterior flange height extended well above the most proximal point of the native trochlear groove. This is typical of most femoral components where the groove on the anterior flange starts well proximal to the groove on the native femur [7,13,14,18,25,26]. This extension of the anterior flange and groove would promote early engagement of the patella with the groove, and a more lateral location of the groove would avoid a tendency for lateral patellar subluxation, particularly in valgus knees with large Q-angles, which is a relatively common complication [1,27,28].

One limitation of this modeling study is that it did not determine whether morphological differences between KA and MA designs are large enough to be clinically important. However, a recent radiographic study [22] using the same KA design as that used herein showed that the line of action of the rectus femoris muscle, which is the preferred line to define the Q-angle [29], was medial to the 20.5° angle of the trochlear groove in all patients. When the line of action of the rectus femoris muscle is lateral to the angle of the trochlear groove, which occurred frequently with the MA design where the groove angle was 6° [22], the patella is medialized, thus increasing the risk of patellofemoral complications. This heightens the interest in future clinical studies which compare outcomes between the KA and MA designs.

Other limitations concern the generalizability of results. For MA, alternative alignment targets exist in both the coronal (e.g., anatomical axis) and axial (e.g., transepicondylar axis) planes [10]. Changing the alignment targets for MA would affect the results. Although the use of alternative alignment targets would affect our numerical results to some degree, it is unlikely that the key findings would be impacted. This is because of the pronounced differences in the trochlear morphology between KA and MA designs (Figure 1).

Next, our results apply only to the component designs studied herein. As other KA designs evolve, it would be worth performing a similar comparative analysis of trochlear morphology to that herein to appreciate differences from the MA design from which the KA design evolved.

Finally, although the sample size was relatively small, it is unlikely that expanding it would affect results. Arguably, the alignment variable that causes the largest difference between MA and KA is the lateral distal femoral angle. This is because this angle determines the difference in varus-valgus alignment between KA and MA for the femoral component. The mean was 87° ± 2° deg with a range of 83° to 89°. This mean is identical to another study with a large sample size (although the range was larger, going from 79.5° to 94.5° [21]). Because the means were the same and because our range was approximately centered in the range of the larger study, the mean difference between KA and MA would be relatively unaffected. Further, although increasing the sample size would increase the variability in differences between KA and MA, at the same time, the larger sample size would decrease the standard error. Moreover, the mean differences were generally highly significant with small *p*-values. For these reasons, it is unlikely that differences in the means determined herein would be markedly affected and that the results of the statistical analyses would change.

## 5. Conclusions

Our results add new information regarding the trochlear morphology of a KA femoral component design. The KA design potentially mitigates the risk of patellofemoral complications by lateralizing and widening the groove, which avoids medializing the patella for wide variations in the lateral distal femoral angle, and by widening the flange laterally to reduce under-coverage. This information enables clinicians to make informed decisions regarding the use of the KA design.

## Figures and Tables

**Table 1:** Repeatability and ICC values for the dependent variables describing the geometry of the trochlea.

	Dependent Variable								
	Radial Distance to Groove	Lateral Height	Medial Height	Lateral Slope	Medial Slope	ML Groove Location	Lateral Peak ML Distance	Medial Peak ML Distance	ML Flat Portion
Repeatability	0.32 mm	0.18 mm	0.32 mm	0.03 mm/mm	0.03 mm/mm	0.54 mm	0.73 mm	0.77 mm	0.93 mm
Intraobserver ICC	0.99	0.74	0.86	0.58	0.33	0.83	0.61	0.76	0.78
Interobserver ICC	0.93	0.65	0.71	0.50	0.28	0.54	0.54	0.71	0.31

## Data Availability

The original contributions presented in the study are included in the article, further inquiries can be directed to the corresponding author.

## References

[1] Assiotis A., To K., Morgan-Jones R., Pengas I.P., Khan W. (2019). Patellar complications following total knee arthroplasty: A review of the current literature. Eur. J. Orthop. Surg. Traumatol..

[2] Barrack R.L., Schrader T., Bertot A.J., Wolfe M.W., Myers L. (2001). Component rotation and anterior knee pain after total knee arthroplasty. Clin. Orthop. Relat. Res..

[3] Conditt M.A., Noble P.C., Allen B., Shen M., Parsley B.S., Mathis K.B. (2005). Surface damage of patellar components used in total knee arthroplasty. J. Bone Joint Surg..

[4] Berger R.A., Crossett L.S., Jacobs J.J., Rubash H.E. (1998). Malrotation causing patellofemoral vomplications after total knee arthroplasty. Clin. Orthop. Relat. Res..

[5] Gonzalez M.H., Mekhail A.O. (2004). The failed total knee arthroplasty: Evaluation and etiology. J. Am. Acad. Orthop. Surg..

[6] Leopold S.S., Silverton C.D., Barden R.M., Rosenberg A.G. (2003). Isolated revision of the patellar component in total knee arthroplasty. J. Bone Jt. Surg..

[7] Saffarini M., Demey G., Nover L., Dejour D. (2015). Evolution of trochlear compartment geometry in total knee arthroplasty. Ann. Transl. Med..

[8] Varadarajan K.M., Rubash H.E., Li G. (2011). Are current total knee arthroplasty implants designed to restore normal trochlear groove anatomy?. J. Arthroplast..

[9] Howell S.M., Papadopoulos S., Kuznik K.T., Hull M.L. (2013). Accurate alignment and high function after kinematically aligned TKA performed with generic instruments. Knee Surg. Sports Traumatol. Arthrosc..

[10] Gu Y., SM H., ML H. (2017). Simulation of total knee arthroplasty in 5 degrees or 7 degrees valgus: A study of gap imbalances and changes in limb and knee alignments from native. J. Orthop. Res..

[11] Gu Y., Roth J.D., Howell S.M., Hull M.L. (2014). How frequently do four methods for mechanically aligning a total knee arthroplasty cause collateral ligament imbalance and change alignment from normal in white patients?. J. Bone Joint Surg. Am..

[12] Dossett H.G., Estrada N.A., Swartz G.J., LeFevre G.W., Kwasman B.G. (2014). A randomised controlled trial of kinematically and mechanically aligned total knee replacements: Two-year clinical results. Bone Joint J..

[13] Lozano R., Campanelli V., Howell S., Hull M. (2019). Kinematic alignment more closely restores the groove location and the sulcus angle of the native trochlea than mechanical alignment: Implications for prosthetic design. Knee Surg. Sports Traumatol. Arthrosc..

[14] Brar A.S., Howell S.M., Hull M.L., Mahfouz M.R. (2016). Does kinematic alignment and flexion of a femoral component designed for mechanical alignment reduce the proximal and lateral reach of the trochlea?. J. Arthroplast..

[15] Rivière C., Iranpour F., Harris S., Auvinet E., Aframian A., Parratte S., Cobb J. (2018). Differences in trochlear parameters between native and prosthetic kinematically or mechanically aligned knees. Orthop. Traumatol. Surg. Res..

[16] Ghosh K.M., Merican A.M., Iranpour F., Deehan D.J., Amis A.A. (2009). The effect of overstuffing the patellofemoral joint on the extensor retinaculum of the knee. Knee Surg. Sports Traumatol. Arthrosc..

[17] Mihalko W., Fishkin Z., Krakow K. (2006). Patellofemoral overstuff and its relationship to flexion after total knee arthroplasty. Clin. Orthop. Relat. Res..

[18] Hull M.L., Howell S.M. (2020). Differences in trochlear morphology from native using a femoral component interfaced with an anatomical patellar prosthesis in kinematic alignment and mechanical alignment. J. Knee Surg..

[19] Bartlett J.W., Frost C. (2008). Reliability, repeatability and reproducibility: Analysis of measurement errors in continuous variables. Ultrasound Obstet. Gynecol..

[20] Indrayan A. (2013). Methods of Clinical Epidemiology.

[21] Hirschmann M.T., Moser L.B., Amsler F., Behrend H., Leclerq V., Hess S. (2019). Functional knee phenotypes: A novel classification for phenotyping the coronal lower limb alignment based on the native alignment in young non-osteoarthritic patients. Knee Surg. Sports Traumatol. Arthrosc..

[22] Sappey-Marinier E., Howell S.M., Nedopil A.J., Hull M.L. (2022). The trochlear groove of a femoral component designed for kinematic alignment is lateral to the quadriceps line of force and better laterally covers the anterior femoral resection than a mechanical alignment design. J. Pers. Med..

[23] Merican A.M., Ghosh K.M., Baena F.R.Y., Deehan D.J., Amis A.A. (2014). Patellar thickness and lateral retinacular release affects patellofemoral kinematics in total knee arthroplasty. Knee Surg. Sports Traumatol. Arthrosc..

[24] Malo M., Vince K.G. (2003). The unstable patella after total knee arthroplasty: Etiology, prevention, and management. J. Am. Acad. Orthop. Surg..

[25] Barink M., Van de Groes S., Verdonschot N., Malefijt M.D.W. (2006). The difference in trochlear orientation between the natural knee and current prosthetic knee designs; towards a truly physiological prosthetic groove orientation. J. Biomech..

[26] Du Z., Chen S., Yan M., Yue B., Wang Y. (2017). Differences between native and prosthetic knees in terms of cross-sectional morphology of the femoral trochlea: A study based on three-dimensional models and virtual total knee arthroplasty. BMC Musculoskelet. Disord..

[27] Lachiewicz P.F., Soileau E.S. (2006). Patella maltracking in posterior-stabilized total knee arthroplasty. Clin. Orthop. Relat. Res..

[28] Lynch A.F., Rorabeck C.H., Bourne R.B. (1987). Extensor mechanism complications following total knee arthroplasty. J. Arthroplast..

[29] Freedman B.R., Brindle T.J., Sheehan F.T. (2014). Re-evaluating the functional implications of the Q-angle and its relationship to in-vivo patellofemoral kinematics. Clin. Biomech..

